# Thermal Tolerance Varies Latitudinally and Broadly Mirrors Genetic Structure in the Seaweed *Phyllospora comosa* Across Its Entire Latitudinal Range

**DOI:** 10.1002/ece3.72720

**Published:** 2025-12-22

**Authors:** Rosalie J. Harris, Callum Bryant, Andrea Leigh, Melinda A. Coleman, Adrienne B. Nicotra, Georgina Wood

**Affiliations:** ^1^ Ecology & Evolution, Research School of Biology The Australian National University Canberra Australian Capital Territory Australia; ^2^ Scripps Institution of Oceanography, Centre for Marine Biodiversity and Conservation University of California San Diego La Jolla California USA; ^3^ School of the Environment University of Technology, Sydney Ultimo New South Wales Australia; ^4^ College of Science and Engineering Flinders University Adelaide South Australia Australia; ^5^ National Marine Science Centre Southern Cross University Coffs Harbour New South Wales Australia; ^6^ New South Wales Fisheries National Marine Science Centre Coffs Harbour New South Wales Australia; ^7^ Oceans Institute and School of Biological Sciences University of Western Australia Crawley WA Australia

## Abstract

Climate‐driven warming is causing rapid changes in marine environments, contracting ranges and reshaping ecosystems. Understanding how genetic structure and phenotypic variation interact to determine populations' ability to tolerate warming aids in predicting biogeographic shifts and informs conservation. We tested whether photosynthetic thermal tolerance in the habitat‐forming seaweed *Phyllospora comosa* reflects underlying genetic differentiation across its full latitudinal range in south‐eastern Australia. We sampled 15 male and 15 female individuals at replicate sites representing three previously defined genetic groups. We measured critical temperature (*T*_crit_) and maximum quantum yield of PSII (*F*_V_/*F*_M_) as metrics of thermal tolerance using temperature‐dependent chlorophyll fluorescence, and assessed relationships with genetic group, sex, latitude and sea surface temperature (SST). We found that thermal tolerance of *Phyllospora* decreased by ~1°C per degree latitude, with low *F*_V_/*F*_M_ in warmer low‐latitude waters, indicating thermal stress. Thermal tolerance patterns loosely mirrored genetic groupings: the warm‐edge group showed the greatest tolerance, while the cool‐edge group was least tolerant. Considerable variation among sites within genetic groups likely reflected both genetic diversity and environmental factors. Males at the warm edge and in central groups tended to show slightly higher tolerance than females, a pattern reversed at cooler latitudes. Although the warm‐edge group showed patterns consistent with local thermal conditions and exhibited higher thermal tolerance, it had poor photosynthetic health when sampled mid‐summer. Geographic patterns of thermal tolerance in *Phyllospora* reflect a combination of genetic differentiation and environmentally driven acclimatory responses, with warm‐edge populations showing high tolerance but reduced diversity and photophysiological health. Central populations which exhibited higher diversity and versatile tolerance may act as a reservoir for restoration. Combining warm‐edge adaptive alleles with central diversity through controlled genetic mixing and outplanting could help future‐proof kelp forest restoration under climate change.

## Graphical abstract

Climate‐driven warming is reshaping marine ecosystems, and understanding how genetic structure interacts with phenotypic thermal tolerance is crucial for predicting biogeographic shifts. Across the full latitudinal range of *Phyllospora comosa*, thermal tolerance declined towards warmer low‐latitude sites and broadly reflected underlying genetic groupings, with warm‐edge populations showing the highest tolerance but poorest photosynthetic health. These patterns suggest that warm‐edge alleles combined with the higher diversity of central populations could support future restoration under climate change.

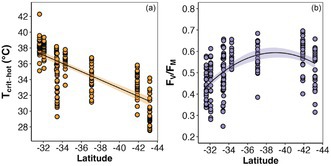

## 1 Introduction

Seaweed forests are intrinsically linked to ecosystem health and function, providing habitat, breeding grounds and nurseries for a plethora of marine organisms (Bennett et al. 2015; Eger et al. 2023; Marzinelli et al. 2014). However, climate change associated ocean warming is pushing seaweeds beyond their physiological limits, causing dieback, range contractions and community reorganisation (e.g., Beas‐Luna et al. 2020; Gurgel et al. 2020; Harley et al. 2012; Smale et al. 2019; Smale and Wernberg 2013; Valentine et al. 2004; Wernberg 2021). Identifying resilient genetic groups that can cope with increased temperatures can inform proactive management and adaptation strategies (Coleman and Goold 2019; Coleman and Wernberg 2020; Wood et al. 2019). However, empirical demonstrations of causation to confirm how such genetic groups perform under thermal stress are rare, but essential to underpin climate interventions.

The ability of seaweed populations to respond to increasing sea temperatures is determined by both their underlying heritable genetic makeup and phenotypic variation (Alsuwaiyan et al. 2021; Kelly 2019; Reed et al. 2011; Veenhof et al. 2024). Genetic variation underpins heritable adaptation, with specific alleles or traits potentially conferring improved performance and survival under thermal stress. Concurrently, individuals can respond to stress with phenotypic plasticity, which, while triggered by environmental factors, is still facilitated by underlying genetic mechanisms. This plasticity is a dynamic response to environmental stimuli, driven by complex molecular and genetic mechanisms (Nicotra et al. 2010). Such plastic responses could provide seaweeds with a significant advantage in adapting rapidly to climate‐induced changes in oceanic conditions.

Understanding the relative importance of underlying genetic variation is important to predict the response of seaweeds to ongoing climate change, but also for the development of proactive management and conservation interventions (Wood et al. 2019; Coleman and Wernberg 2020). For example, assisted adaptation (the intentional movement or selection of individuals with favourable traits to enhance the adaptive potential of populations facing environmental change [Breed et al. 2019]) and assisted evolution (human intervention to accelerate the natural evolutionary processes of organisms [van Oppen et al. 2015]) depend heavily on a comprehensive understanding of genetic diversity within a species because its intended outcome is that traits are perpetuated/inherited across generations. Identification of populations containing individuals with high frequencies of loci under putative selection to higher sea surface temperatures (SST) can be a starting point for such strategies because it is likely that such individuals have a greater ability to withstand thermal stress and that this will be perpetuated across subsequent generations (Wood et al. 2024). While a relatively nascent area, some recent population genetic studies point to such putatively adaptive loci in seaweed populations (e.g., Minne et al. 2025; Vranken et al. 2021; Wood et al. 2021); however, these genetic patterns are yet to be confirmed through empirical physiological studies that demonstrate causation linking genetics with performance.

A common metric to assess the response of photosynthetic organisms, including seaweeds, to thermal stress are measures of maximum efficiency of photosystem II (*F*_V_/*F*_M_) in the photosynthetic tissue. Photosystem II (PSII) is a protein‐pigment complex in chloroplasts that plays a key role in photosynthesis. PSII absorbs light energy to initiate the conversion of water and carbon dioxide into oxygen and sugars, producing chemical energy in the form of ATP and NADPH (Barber 2006). *F*_V_/*F*_M_ is a direct measure of the photosynthetic efficiency and health of an individual seaweed and indicates how well PSII can convert light energy into chemical energy, which is essential for seaweed growth and survival (Maxwell and Johnson 2000). The expectation of a consistent *F*_V_/*F*_M_, coupled with variations in critical thermal limits, assumes a relationship between genetic predisposition and environmental responsiveness. Seaweeds, like many other organisms, can adjust their physiology to cope with varying environmental conditions (Pereira et al. 2015). This acclimation is a form of plasticity, allowing organisms to maintain optimal functioning despite changes in their surroundings. This might enable seaweeds to maintain healthy photosynthetic carbon gain (ability to obtain and utilise carbon dioxide for growth and metabolic processes), whilst ensuring their role in marine ecosystems amidst fluctuating temperature conditions (Werner et al. 2001). However, it is not known whether thermal tolerance within PSII of seaweeds is a result of acclimation to immediate environmental conditions or a more fixed, genetically determined limit. The innate reaction norm for this trait could remain consistent across latitudes, but its manifestation under varying environmental conditions might differ. This perspective aligns with the understanding that plastic responses can be crucial in short‐term acclimation to changing climates, setting the stage for longer‐term evolutionary adaptations.

To measure the temperature response of photosynthetic organisms, a well‐established approach in the field of terrestrial plant ecophysiology is that of the temperature‐dependent fluorescence (*T*‐*F*_0_) method (Ilík et al. 2003; Schreiber and Berry 1977). This method allows for high throughput sampling to identify individuals' sensitivity within the thylakoid membrane of PSII and acts as an indicator of thermal stress when temperatures are progressively increased (Arnold et al. 2021). Heat stress can impact photosynthesis by damaging PSII, essential for chloroplast electron transfer (Bukhov et al. 1999). This may involve the loss of manganese ions from PSII's oxygen‐evolving complex (Enami et al. 1994). The condition of PSII has become a standard gauge of photosynthetic heat tolerance commonly used (Atwell 1999; Knight and Ackerly 2002; O'sullivan et al. 2013; Geange et al. 2021). Employing this technique has revealed important insights into global patterns of plant thermal tolerance (Lancaster and Humphreys 2020). For instance, comparative studies using this method among species or populations from different thermal biomes point to evolutionary adjustments in photosynthetic thermal stability (Knight and Ackerly 2002; Zhu et al. 2018). Furthermore, in the realm of agriculture, chlorophyll fluorescence has become instrumental in pinpointing crop genetic groups with heightened thermal tolerance, thereby informing breeding strategies aimed at enhancing resilience to temperature extremes (Coast et al. 2021; Posch et al. 2019). A study recently revealed that this method could be utilised in the field of thermal physiology in seaweeds; however, it has yet to be used to investigate spatial and temporal patterns in critical temperatures (Harris et al. 2022).

Along the south‐eastern coast of Australia, shallow seaweed forests are often dominated by the dioecious habitat‐forming fucoid *Phyllospora comosa* (C. Agardh), hereafter referred to as *Phyllospora* (Coleman and Wernberg 2017). This endemic species stretches across a latitudinal gradient of 31°–43° accompanied by substantial variation in SST of 12°C–26°C and inhabits mainland Australian waters up to 6 m in depth, though in Tasmania it can be found at depths extending to 18 m (Coleman et al. 2008; Wood et al. 2021). Along the east Australian coastline, *Phyllospora* forms three genetically distinct groups (Wood et al. 2021): a warm water northern group, a large and highly connected central group, and a cool‐water southern group. Previous genetic analysis of single nucleotide polymorphism (SNP) data has indicated the presence of genetic‐environmental associations with SST, with populations at the warm and cool range edges possessing low genetic diversity but putative adaptation to local environmental conditions; however, this correlation remains to be validated with experimental or functional data. This work also found biases in population sex ratios (male bias) at the warm edge, within the warm water genetic group, potentially suggesting that this bias may have come about due to differences in thermal tolerance between the sexes (Edmands 2021; Valeria Oppliger et al. 2011).

Here, we explored the links between the genetic structure and ecophysiology of *Phyllospora*, specifically thermal tolerance (measured as the critical temperature [*T*_crit_] at the onset of stress within the thylakoid membrane) across the entire latitudinal range of the species. We conducted thermal tolerance assays at replicate sites based on the sites from Wood et al. (2021) representative of each genetic group previously identified by SNP analysis to investigate patterns of thermal tolerance. Specifically, we posited that (1) sites with higher SST would have higher thermal tolerances compared to those with lower SST; (2) if photochemical efficiency measured by *F*_V_/*F*_M_ remained constant across the gradient, it was expected that the variation in thermal tolerance (critical temperature measured as *T*_crit_) would support a stable rate of carbon assimilation because *F*_V_/*F*_M_ reflects baseline PSII photochemical efficiency in dark‐adapted tissue and is generally stable unless chronic stress is present. In contrast, *T*_crit_ represents the acute temperature threshold at which PSII becomes thermally damaged and is highly responsive to thermal history and membrane stability; (3) if thermal tolerance was determined by genetics, patterns of genetic clustering would align with patterns of thermal tolerance, such that genetic groups with loci under selection are differentiated by thermal tolerance and (4) if the skewed male‐dominant sex ratios in the warmer range edge group reflected sex‐based differences in thermal tolerance for this species, then male plants might exhibit a higher *T*_crit_ than female plants in warmer locations. By linking metrics of thermal tolerance to genomics, we aim to provide insight into the various mechanisms underpinning adaptation to warming in a key habitat‐forming seaweed and identify target lineages for future‐proofing restoration efforts.

## 2 Materials and Methods

### 2.1 Site and Species Selection

Site selection was based on the genomics work of Wood et al. (2021). We sampled replicate sites within each of the three genetic groups of *Phyllospora* to link thermal tolerance metrics to the site‐level genomics work (Figure 2). The three genetic groups were a northern group linked to warm‐water associated loci under selection, a central group, and thirdly, a southern cool‐water associated group with loci under selection linked to cooler temperatures. Samples were collected on snorkel at low tide each day during the warmest time of the year to assess their field‐acclimated physiological state. Sampling occurred from 18 February to 4 March 2022, starting at the most northerly genetic group in Port Macquarie NSW and ending in Port Arthur, Tasmania Australia. At each site, we sampled 15 males and 15 females and conducted thermal tolerance assays (described below). During sample collection, sea‐water temperature at a depth of 2 m was measured at 30 s intervals for 20 min at each site using a HOBO data logger to capture an estimate of SST at the time of collection (HOBO UX120; Onset). Temperatures were averaged for each site to provide a measure of sea surface temperature (SST), which was strongly correlated with latitude as expected (*R* ^2^ = 0.86 between SST and latitude, Figure S1). Water depth of sample collection (~2 m), time of day, and tide were kept consistent throughout sampling. Upon collection, samples were placed in wet cotton bags in a cooler to maintain turgor, transported back to a mobile laboratory, and thermal tolerance measurements were carried out within 1 h from the time of collection.

### 2.2 Thermal Tolerance Assays

In assessing thermal tolerance across individuals, species or genetic groups, *T*_crit_ must be interpreted as a relative, rather than an absolute value. This is a cellular response, not an organismal death point, hence why the values are always higher than the SST. It is a measure of stress, not survival, and thus serves as an early warning indicator of thermal stress; thus, this perspective ensures a more realistic interpretation of *T*_crit_. We conducted temperature‐dependent fluorescence (*T*‐*F*_0_) using methods from Harris et al. (2022) to extract critical thermal tolerance temperatures (*T*_crit_) for each sample. This method involved measuring changes in basal chlorophyll fluorescence (*F*_0_) in response to a steady increase in temperature. This method involved measuring the thermal rise in chlorophyll fluorescence that occurs when PSII reaction centres begin to incur heat damage during a controlled temperature ramp. *F*_0_ is the minimal fluorescence of dark‐adapted tissue, *F*_M_ the maximal fluorescence following a saturating pulse, and *F*_V_ = *F*_M_ − *F*_0_ represents variable fluorescence, a measure of PSII photochemical capacity.

Samples were first cut into 1 × 1 cm^2^ sections using a razor blade and then placed onto a gridded paper array of 30 samples and covered with cling film to avoid desiccation. Sample arrays were placed onto a Peltier block with a thermocouple placed on top of every sample, and dark adapted for 20 min. Initial maximum quantum yield of PSII (*F*_V_/*F*_M_) was taken prior to the assay to ensure individuals were healthy. The heating Peltier plate was set to increase at 15°C h.^−1^, starting at the local seawater temperature as measured and ramped to 50°C, taking about 2 h to complete. Basal fluorescence was measured every 20 s over the course of the assay using a weak modulating blue light that is too low to drive photosynthesis. Temperature was recorded every 5 s by a dataTaker DT85 (Lontek, Glenbrook, New South Wales, Australia). Values of *T*_crit_ were extracted following the protocols by Harris et al. (2022), whereby the breakpoint from the temperature‐dependent fluorescence curve was determined by fitting lines to both the slow and fast phases of the curve. This breakpoint is the temperature at which the onset of stress is notable within the thylakoid and is used as a proxy indicator of thermal tolerance.

### 2.3 Linking Genomic Data With Thermal Tolerance

We used overall loci—including both neutral and putatively adaptive SNPs—for genetic clustering and group assignment, consistent with Wood et al. (2021). This decision was based on two factors: first, the number of loci under selection was limited and thus genetic diversity measures were not calculated, and second, patterns of genetic structure derived from the outlier loci were broadly concordant with those obtained using the full SNP dataset. Using overall loci also allowed us to maintain sufficient resolution for clustering across all individuals and sites, facilitating robust comparisons of thermal tolerance across genetically defined groups.

We paired population‐level measures of genetic diversity to thermal tolerance physiology (coefficient of variation of *T*_crit_) using three key indices: genetic diversity (HE), allelic richness (AR) and inbreeding (F_IS_) to help understand the relationships between genetic diversity and thermal tolerance. Expected heterozygosity (HE) is the (expected) probability that an individual will be heterozygous at a given locus and therefore represents average population‐level genetic diversity (Nei 1973; Riquet et al. 2021). Allelic richness represents the number of different alleles in a population and is a key factor in adaptability, providing a diverse genetic pool for selection under varying thermal stresses. Finally, inbreeding measures genetic similarity and its associated reduction in diversity, impacting a population's ability to adapt and endure thermal stress (Erard 2004). By correlating these indices with the coefficient of variation of *T*_crit_, we explored whether groups with higher genetic diversity, allelic richness, and lower inbreeding exhibited a greater variation in thermal tolerance responses. A high CV of *T*_crit_ indicates a wide range of thermal tolerance within a group and could imply a high degree of phenotypic variation, potentially linked to diverse genetic backgrounds. Conversely, a low CV of *T*_crit_ signifies that the thermal tolerance is more uniform across the population, which might indicate genetic homogeneity or limited phenotypic plasticity. If genetic diversity directly underpins thermal tolerance, populations with higher genetic diversity would be expected to show greater variability in thermal tolerance responses due to a broader range of individual genetic makeups.

### 2.4 Statistical Analysis

All analyses were performed in R version 4.0.2 (R Core Team 2018) using the Car package and lmerTest package. We used a linear mixed‐effect model to assess the different genetic groups and whether *F*_V_/*F*_M_ and *T*_crit_ varied between sexes. In these models, *T*_crit_ was the response variable, genetic group and sex were fixed effects and site was a random effect nested in the genetic group. Due to finding a high degree of variation explained by site in the linear mixed‐effects models (model fit improved from 0.4 to 0.8 with site), we also explored how *T*_crit_ varies across sites with site nested within the genetic groups.

Initial analyses revealed that time of day, depth and tide did not meaningfully account for any of the variation and so were not included in any of the final models. Because sea surface temperature and latitude were highly correlated (*R* ^2^ = 0.86, Figure S1) and yielded statistically identical model results, we report latitude as the main predictor for clarity and to emphasise geographic structuring across the range of *Phyllospora*. To address our hypothesis that thermal tolerance was related to ambient SST, rather than genetic identity, we used a linear model to test for correlations between *T*_crit_ (response) and latitude (fixed effect). To test for uniformity of *F*_V_/*F*_M_ across latitude, we used a similar model but with a second‐degree polynomial regression as it explained substantially more variance than a linear model. In addition, we examined correlations between our measures of population genetics (HE, AR and F_IS_) and variance in *T*_crit_ values by plotting means from the genetic indices for each site by the coefficient of variation (CV, stdev/sqrt[mean]) of *T*_crit_ and fit a simple correlation post hoc to assess the fit of these associations.

## 3 Results

**FIGURE 1:**
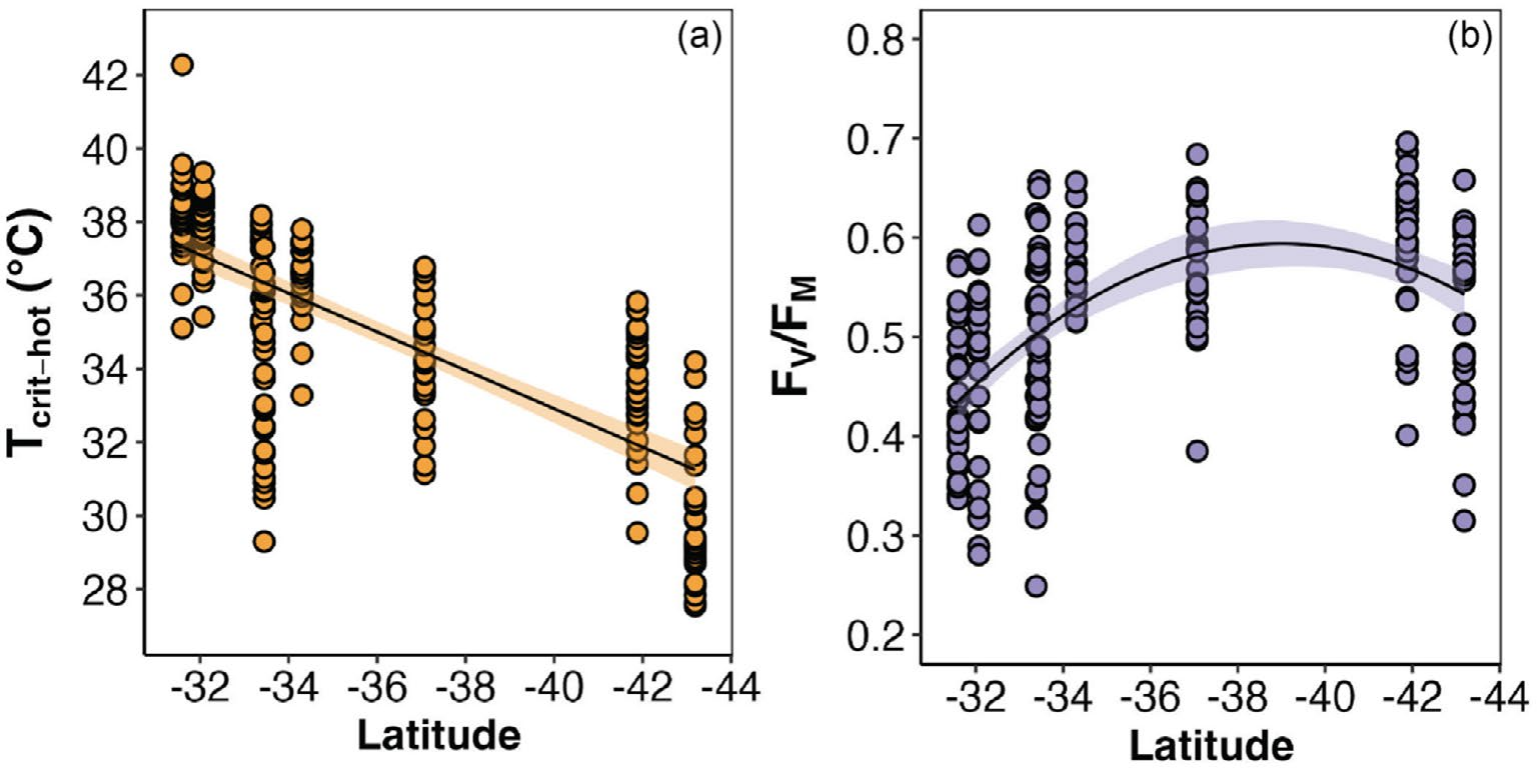
Photosynthetic thermal tolerance metrics for *Phyllospora* across latitude. (a) Raw values of *T*_crit_ over the latitudinal gradient with a fitted linear regression showing strong declines in thermal tolerance with increasing latitude. (b) Effective quantum yield (*F*_V_/*F*_M_) over latitude with a polynomial model fit, depicting lowest photosynthetic health towards the range edges—particularly the warm lower latitude group—and then highest values in the mid‐ranges.

Thermal tolerance was negatively correlated with SST across the latitudinal range of *Phyllospora* (*F*_1_ = 243, *p* < 0.001, Figure 1a, Table 1). These values were consistently about 11°C–12°C above the in situ SST. *T*_crit_ exceeded SST at every location, with an average *T*_crit_ of 38°C compared to SST of 27°C (as measured in situ at the time of sampling) in the warmest range edge site. As for the coolest range edge, *T*_crit_ was 30°C and an SST of 18°C. Maximum quantum yield of PSII (*F*_V_/*F*_M_) (i.e., an instantaneous measure of plant photosystem health at the time of sampling) also varied significantly, but non‐linearly, with latitude (*F*_2_ = 40.132, *p* < 0.001, Figure 1b, Table 1), reaching its optimum in the central genetic group, whilst the northern and southern genetic groups had lower *F*_V_/*F*_M_ values, particularly in the warm edge genetic group (Figure 1b).

**TABLE 1:** ANOVA results from two different linear models to determine variation of *T*_crit_ and *F*_V_/*F*_M_ across latitude.

Response	Sum Sq	df	*F*	*p*
*T*_crit_	630.63	1	142.07	< 0.001
*F*_V_/*F*_M_	0.521	2	50.922	< 0.001

**FIGURE 2:**
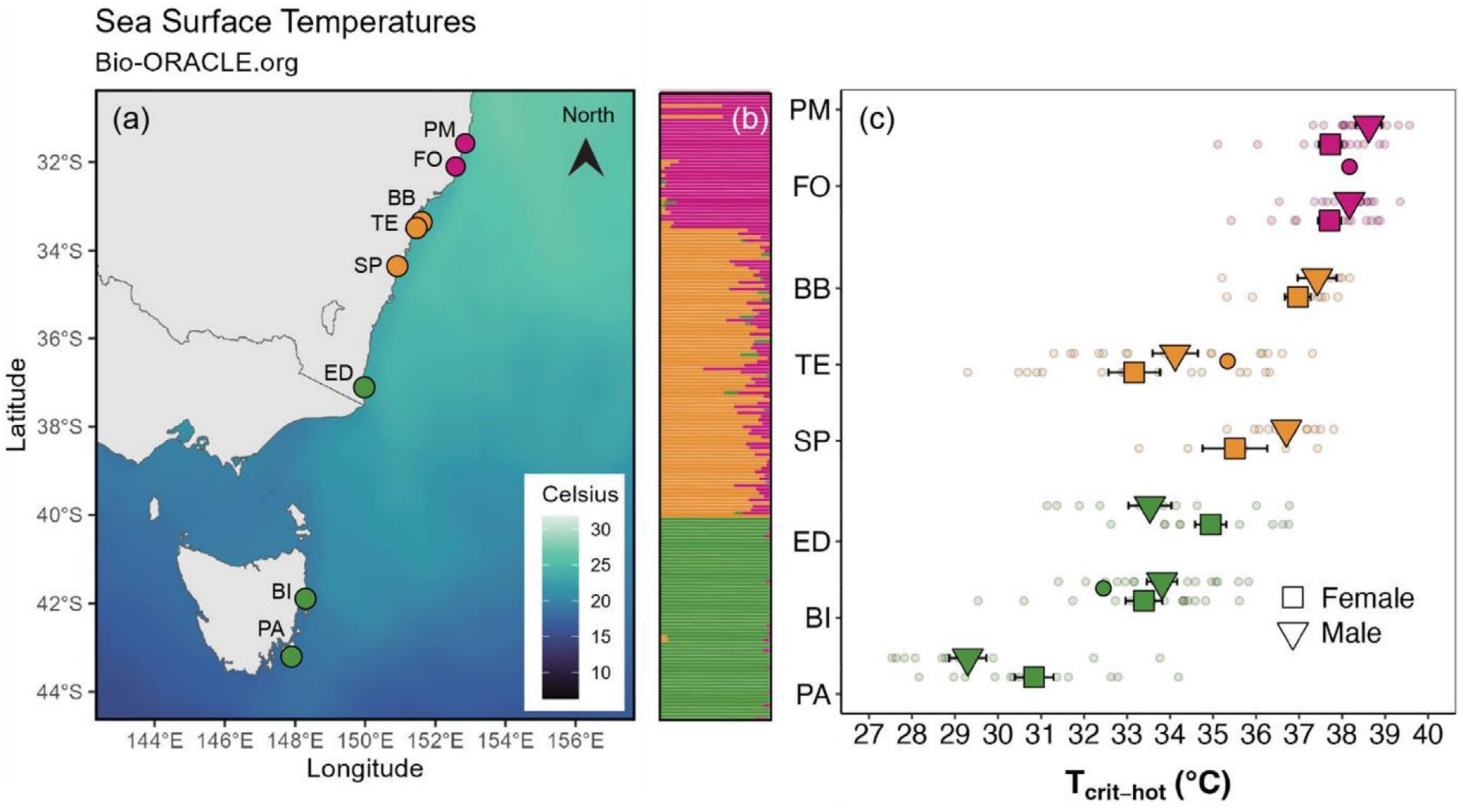
Patterns of thermal tolerance of *Phyllospora* for different genetic groups. (a) Sea surface temperature (mean maximums) for each site during time of sampling in mid‐February 2022 with coloured site points matching genetic groups; (b) genetic structure plot modified from Wood et al. (2021) showing three genetic groups based on all (neutral and putatively adaptive) loci and (c) thermal tolerance (*T*_crit_) means and standard errors for genetic groups (coloured circles) and site (triangles and squares—males and females, respectively) with colour matched to the genetic structure plot.

There was a significant two‐way interaction between sex and genetic group for thermal tolerance across latitude (Figure 2, Table 2). Post hoc tests revealed significant differences between sexes within the central and southern groups, with males having a higher thermal tolerance than females in the central genetic group and females being higher than males in the southern genetic group (see Table 3 for pairwise comparisons).

**TABLE 2:** ANOVA results from a linear mixed‐effects model to determine if *T*_crit_ varied with genetic group and sex.

Predictors	*F*	df	*p*
Genetic group	4.148	3	0.102
Sex	0.479	1	0.489
Sex × genetic group	4.944	3	**0.002**
Marginal *R* ^2^: 0.471			
Conditional *R* ^2^: 0.851			

*Note:* Analysis of deviance table (Type II Wald *F* tests with Kenward–Roger df). Bold indicates significance at *p* < 0.05, *p* < 0.01, *p* < 0.001.

**TABLE 3:** Post hoc comparisons of the differences in *T*_crit_ for female and male *Phyllospora* for each genetic group based on a significant interaction between sex and genetic group from the linear mixed‐effects model as shown in Table 2.

Genetic group	Δ*T*_crit_ estimate between female and male	SE	df	*t*‐ratio	*p*
Northern:	−0.686	0.391	189	−1.752	0.081
Central:	−0.892	0.391	190	−2.282	**0.023**
Southern:	1.413	0.609	189	2.322	**0.021**

*Note:* Bold indicates significance at *p* < 0.05, *p* < 0.01, *p* < 0.001.

**FIGURE 3:**
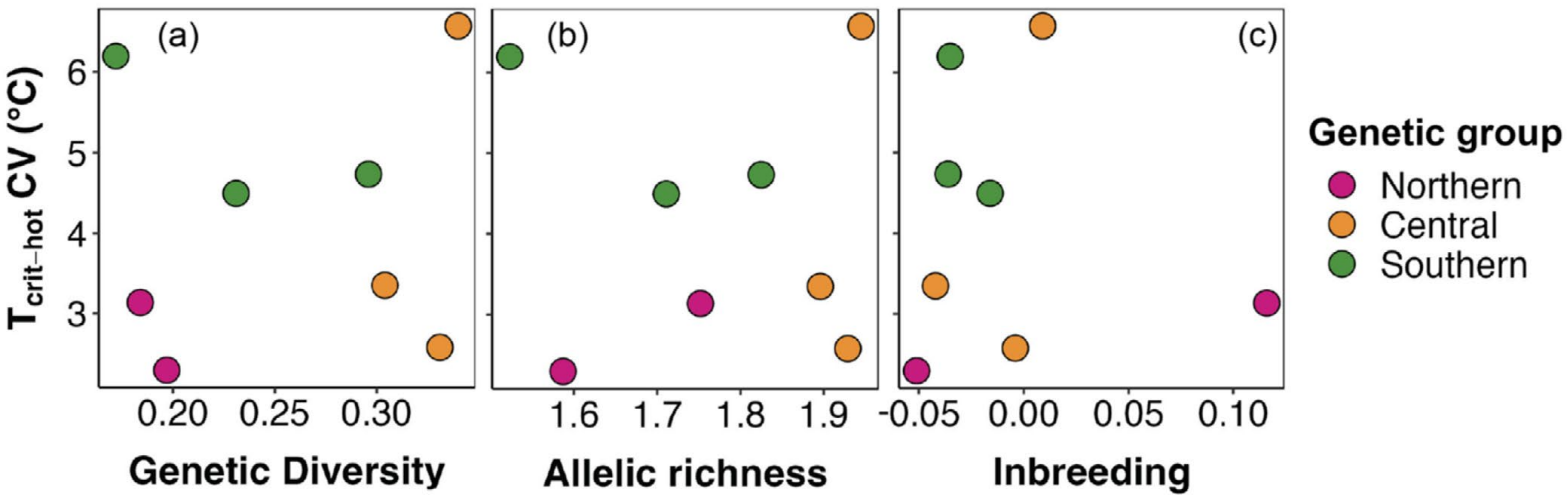
Coefficient of variation of *T*_crit_ (which quantifies the extent of variation in *T*_crit_ values within the means from each site for each genetic group) and (a) genetic diversity (*H*_e_), (b) allelic richness and (c) inbreeding (*F*_IS_) for *Phyllospora*. Coloured points depict genetic groups matched with the colours from the genetic structure plots from Figure 2. The current study included two to three sites per genetic group that matched original sites from Wood et al. (2021).

We assessed how patterns of thermal tolerance were linked to genetic clustering. Variation in *T*_crit_ with latitude/SST displayed more of a gradient as opposed to distinct clustering with the genetic groups (Figure 2). However, it did show that the warm water associated genetic group had the highest thermal tolerance and those in the south had the lowest (Figure 2). When we visually examined whether measures of genetic diversity within sites were correlated with the coefficient of variation of *T*_crit_, we found that there was no association (Figure 3a,b); likewise, there was also no association between inbreeding and *T*_crit_ CV (Figure 3c).

Finally, we assessed differences between/among sites within genetic groups in variation of *T*_crit_. Although there was an almost 10° difference between the warm range edge in Port Macquarie (PM) and the cooler range edge in Port Arthur (PA), there was no significant difference in variation of *T*_crit_ between/among sites within genetic groups (Table S1).

## 4 Discussion

Our investigation into the thermal tolerance of the habitat‐forming seaweed *P. comosa* across its entire latitudinal range in Australia revealed key insights linking photosynthetic thermal tolerance to both sea surface temperature (SST) and genetic structure. Thermal tolerance was tightly associated with the latitudinal gradient in SST and broadly mirrored the species' known genetic structure, but there was substantial within‐group variation, indicating that local thermal history and acclimatory responses may also explain variation in *T*_crit_ beyond genetic grouping. Critically, however, thermal tolerance was not uniformly reflected in capacity for carbon gain, as indicated by non‐uniform *F*_V_/*F*_M_ values across the gradient. In the warm‐edge group, despite high *T*_crit_, the notably low *F*_V_/*F*_M_ values suggest a physiological trade‐off between maintaining PSII thermal stability and sustaining maximal photochemical efficiency. Thus, individuals in the warm‐edge group may tolerate extreme heat without immediate PSII failure, but at the cost of reduced photochemical efficiency and carbon assimilation. However, the relationship between genetic grouping and thermal tolerance likely reflects shared responses to the latitudinal SST gradient rather than direct genetic control and disentangling these effects will require future common‐garden or reciprocal transplant experiments.

There was also a subtle trend (non‐statically significant) for males in the warm edge genetic group—an area previously found to have a male‐bias at the furthest warm edge site—to exhibit higher thermal tolerance, and females showed significantly higher tolerance in the southern leading‐edge group. Finally, although central populations harbour greater genetic diversity and robust photophysiology, they lack some of the putatively warm‐adapted alleles found in the warm edge group (Wood et al. 2021). This creates a restoration challenge: although central populations harbour greater genetic diversity, resilience to thermal stress likely depends on the presence of locally adapted, warm‐tolerant alleles rather than diversity alone. However, our findings also suggest that some populations may cope with warming through acclimatory responses, particularly if temperature increases are gradual. Thus, successful restoration will likely require a dual approach, combining the genetic breadth of central populations with locally adapted alleles from the warm edge. Specifically, combining the broader diversity of central populations with the putatively warm‐adapted alleles from range‐edge groups through controlled cultivation and outplanting experiments has been proposed as a potential strategy, as such crosses may allow recombination of beneficial traits and increase resilience under future climate scenarios. However, whether the warm‐edge differences observed here have a genetic basis remains untested, and any mixing of populations would require careful evaluation to avoid disrupting locally adapted gene complexes or causing outbreeding depression. In addition, this study cannot determine whether the observed thermal tolerance differences have a genetic basis or instead reflect environmentally driven acclimatory responses. Demonstrating a heritable component would require future common‐garden, breeding, or reciprocal transplant experiments.

### 4.1 Latitude, Sea Surface Temperature, Light and the Variation of Thermal Tolerance in *Phyllospora*

Across the 10°C difference in SST along the eastern latitudinal range of *Phyllospora*, *T*_crit_ differed by 10°C between the warmest and coolest range edges. This result corroborates the notion that the critical temperature of photosystem II (PSII) is highly variable. This variation in *T*_crit_ may indicate that *Phyllospora* can substantially modulate this critical threshold in alignment with changing thermal conditions, a not so surprising result given similar findings from the terrestrial literature. For example, O'Sullivan et al. (2017) demonstrated considerable species‐level variation in *T*_crit_ among terrestrial plants, with observed ranges extending from 41.5°C in the Alaskan arctic to 50.8°C in the Peruvian Amazon, reflecting diverse adaptations along latitude. Within Australian plants, *T*_crit_ not only varies with the plant's native habitat but also acclimates to current growth temperatures (Zhu et al. 2018). These findings illustrate that variation in *T*_crit_ among terrestrial plants along latitudinal gradients, as well as possibly for *Phyllospora* here, is a combination of inherent trait differences, genetics and acclimation. We found an upper *T*_crit_ of 38°C in the warm edge group, reflecting the onset of stress within the thylakoid at SSTs of ~27°C, which is the maximum annual temperature currently experienced across the species' range (Coleman and Wernberg 2017). To determine the extent to which this acclimation capacity can protect against higher SSTs, future research should aim to elucidate the absolute upper limit of *T*_crit_ in *Phyllospora* to increases in SST. Note that while *T*_crit_ generally increased towards lower latitudes, latitude here is used as a spatial descriptor that broadly reflects, but is not perfectly interchangeable, with the SST gradient. Mesoscale processes such as the seasonal strengthening of the Eastern Australian Current and local upwelling can create fine‐scale thermal anomalies (Ridgway and Hill 2009) that may partly explain within‐group variability not captured by mean SST values.

Beyond the effects of ambient thermal history profiles, light and nutrients play an important role in shaping the thermal tolerance of *Phyllospora*, such as recent work showing that juvenile *Phyllospora* exposed to moderate light levels experienced transient reductions in photosynthesis under heatwave conditions but demonstrated rapid recovery, potentially via physiological adjustments in membrane lipids (Britton et al. 2020). Given that PSII is directly involved in the light‐harvesting process for photosynthesis, variations in light intensity (both along latitudes and depths) interact with temperature to affect the overall health of the photosynthetic apparatus (Eggert 2012). Specifically, lower light intensities experienced by *Phyllospora* at depth or higher latitudes could contribute to its ability to tolerate lower temperatures on the cool range edge, whereas higher irradiance levels in shallower or warmer regions might lead to increased energy demand and heightened susceptibility to photoinhibition at elevated temperatures. Previous work has shown that thermal stress can amplify photoinhibition under high light, while moderate light may buffer against temperature extremes (Gao et al. 2017).

### 4.2 Thermal Tolerance and Genetic Homogenisation at the Warm Range Edge

We found that sites within the warm range edge genetic cluster had both low genetic diversity and low coefficient of variation (CV) in thermal tolerance, which is consistent with two interpretations: (i) that this group has undergone local adaptation (selection), optimising their thermal tolerance to higher SST experienced in the area and/or (ii) this group has acclimated to higher temperatures via possible plastic mechanisms. Although *F*_V_/*F*_M_ quantifies PSII efficiency rather than carbon fixation directly, it is a well‐established proxy for potential photosynthetic performance, as it correlates strongly with carbon assimilation and growth in algae (Hanelt 1996; Raven and Geider 1988). Given the low *F*_V_/*F*_M_ which indicates that the populations are at their thermal margins, we suggest that their ability to resist high temperatures is a result of environmentally induced responses, rather than genetic adaptation. However, this might also reflect either a demographic bottleneck effect, where selective pressures (and subsequent low genetic diversity) favour specific responses for survival, such as higher *T*_crit_, or an inherent physiological or genetic trade‐off between these traits, where increasing PSII thermal stability constrains maximal photosynthetic, as indicated by low *F*_V_/*F*_M_ values. Such a scenario would imply that while the response of a high *T*_crit_ may aid in surviving warmer temperatures, it could concurrently compromise photosynthetic productivity, a critical factor for the long‐term survival of this warm edge group. Disentangling the relative roles of genetics, environment, and genotype‐by‐environment effects in driving these differences will require targeted common‐garden and reciprocal‐transplant experiments.

As these seaweeds adapt to tolerate higher temperatures, they may need to redirect energy and resources towards maintaining cellular integrity and stress responses, which are needed for survival in harsher thermal environments (Leles and Levine 2023). This reallocation of resources, while beneficial for withstanding temperature extremes, could result in less energy being available for photosynthesis (Hemme et al. 2014; O'Donnell et al. 2018). This is further supported by the incredibly low photosystem health status represented by *F*_V_/*F*_M_ values around 0.4 in the warm edge genetic group. Although high *T*_crit_ values are typically interpreted as indicative of robust thylakoid stability, their co‐occurrence with reduced *F*_V_/*F*_M_ suggests a physiological trade‐off between maintaining PSII thermal tolerance and sustaining maximal photochemical efficiency. Populations experiencing chronically high temperatures may invest more heavily in mechanisms that stabilise or repair PSII, such as thylakoid membrane restructuring or synthesis of heat‐shock proteins, thereby elevating *T*_crit_ but at the cost of baseline PSII performance. Similar trade‐offs have been demonstrated experimentally in microalgae adapted to elevated temperatures, where increased PSII thermal tolerance was associated with reduced photosynthetic efficiency and growth (Jin and Agustí 2018). Functionally, this compromise could constrain carbon assimilation (Raven and Geider 1988). And under further warming of 2°C–4°C, may accelerate the decline of individuals in the warm‐edge group (Diehl et al. 2021; Martínez et al. 2018), particularly with gradual warming of SSTs and under the increased frequency and intensity of marine heatwaves due to climate change (Oliver et al. 2018). These findings underscore the need to understand physiological trade‐offs in seaweeds, which may ultimately determine their resilience or vulnerability to ongoing climatic shifts (Boyd et al. 2016; Coleman and Wernberg 2021).

In contrast, the central group of *Phyllospora* demonstrated higher genetic diversity compared to other regions, accompanied by a low coefficient of variation (CV) in *T*_crit_. We note however, that this pattern is not uniform across all central populations: while two sites show low CV, one central population exhibits the highest CV observed, highlighting within‐region heterogeneity in thermal performance. This could imply that the central group have established a thermal tolerance range that is universally suitable across the genetically diverse individuals within this region, alternatively, it might signify a high degree of plasticity in *T*_crit_ (i.e., a non‐genetic underpinning) among the diverse genetic groups present in this group, either way, both are responding to local SST (Mohring et al. 2014; Nilsson‐Örtman et al. 2012). The central group, which seems to be thriving in areas with mild to warm sea surface temperatures are likely inhabiting their thermal optimum as evidenced by robust photosynthetic health with high overall *F*_V_/*F*_M_ values. This diversity may act as a reservoir for potential adaptation to future environmental changes (Somero 2010) given that diversity is generally known to allow a genetic group to respond to changing environmental conditions (Allendorf et al. 2012; Wernberg et al. 2018). While all genetic groups possess distinct allele compositions, the edge populations harbour SST‐associated alleles that reflect adaptation to their local thermal environments (Wood et al. 2021). These alleles reflect local adaptation to extreme conditions but are found in groups with low genetic diversity, potentially limiting their longer‐term adaptive capacity. In contrast, the central populations possess greater genetic diversity and robust thermal performance but lack these specific SST‐adapted variants. Future‐proofing *Phyllospora* will therefore require integrating the genetic diversity of central populations with the locally adapted alleles from the warm edge, and thus combining breadth and specificity to maximise resilience under ongoing ocean warming.

### 4.3 Sexual Dichotomies in Thermal Tolerance

In dioecious species, variation in sex ratios can be an indicator of differences in survival and physiological tolerance, particularly at a range edge (Delph 1999; Valeria Oppliger et al. 2011; Viejo et al. 2011). Given that Wood et al. (2021) found a significant male sex ratio bias in the warm edge genetic group, we hypothesised that males had a higher inherent heat tolerance than females (Liu et al. 2017). Although we did not find major differences in thermal tolerance between the two sexes across the latitudinal gradient, there was a non‐significant trend for higher tolerance in males in warm edge and central genetic groups, and in females towards the leading‐edge group. When we accounted for genetic structure, there were differences in thermal tolerance between males and females in the central genetic groups (male higher) and those within the southern groups (females higher); although this did not explain the sex bias, it did suggest that sex matters regarding thermal performance. Differences in sex ratios and physiological responses across populations often have profound ecological implications (Bürli et al. 2022; Field et al. 2013; Nicotra 1998). Discrepancies in thermal tolerance between males and females could impact various aspects of a species' life history, such as reproduction and survival rates. For instance, if males are more heat tolerant, they might have a survival advantage in warmer climates, potentially leading to further skewed sex ratios. Other fucoid species have been impacted by warming temperatures that can disrupt the timing of reproduction and alter early life history stages (Coleman and Brawley 2005). The central and leading‐edge groups show variation in how males and females respond to environmental conditions when we consider the genetic structure of these groups. The predominance of males in the warm edge group hints at potential reproductive challenges. The energy‐intensive task of egg production could result in females facing greater physiological stress compared to males, potentially leading to reduced reproductive success or greater female mortality. Regardless, the sex bias at the range edge and trend for differences in thermal tolerance between the sexes warrants further research.

### 4.4 Adaptive Responses and Future Trajectories

Our findings highlight the vulnerability of warm‐edge *Phyllospora* populations, which exhibit high thermal tolerance and SST‐associated alleles, but also low genetic diversity and impaired photophysiological performance. In contrast, the central group maintains high genetic diversity and relatively stable *F*_V_/*F*_M_ values, suggesting greater resilience and canalization under current conditions. However, their lack of warm‐adapted alleles may leave them vulnerable to future ocean warming. This reinforces the importance of combining locally adapted genotypes from the warm edge with the genetic breadth of central populations to maximise adaptive potential. This concept aligns with King et al. (2019) and Wernberg et al. (2018), who demonstrated that local adaptation in seaweeds can lead to the development of narrower thermal tolerance ranges. This adaptation is crucial as it allows species to tune their responses to their immediate environments, a process that can be especially significant in areas where a species' thermal niche is likely to be exceeded first, such as at range edges.

As ocean temperatures rise, it is predicted that many key seaweeds will increasingly contract their ranges poleward (Davis et al. 2022; Martínez et al. 2018) and that genetic adaptation will not keep pace with climate change (Vranken et al. 2021; Minne et al. 2025; Wood et al. 2021). While deeper waters might offer a cooler refuge, *Phyllospora*'s limitation to waters up to 6 m deep constrains this possibility (Cheung et al. 2009; Davis et al. 2021; Pereira et al. 2015). Instead, our data suggest a ‘squeeze’ scenario, with the species' depth limit constrained by light requirements. Given that poleward migration is not a viable option, this species will instead experience significant range contractions. The central genetic group, with its high genetic diversity, could act as a genetic reservoir for *Phyllospora*'s recovery, especially if the warm edge group declines (Coleman and Wernberg 2020). While selecting adaptive alleles from the warm edge group could enhance the central group's thermal tolerance (Wood et al. 2021), our findings show that *T*_crit_ values in the central group are as high as those in the northern group. This underscores the need for common garden experiments to disentangle genetically determined trait variation from environmental effects, providing clearer insights into the heritability of thermal tolerance traits. Importantly, changes in *Phyllospora*'s distribution are likely to have profound effects beyond the species itself, influencing the structure and dynamics of the marine communities they support (Marzinelli et al. 2014, 2016; Wood et al. 2022). Proactive monitoring and adaptive management of *Phyllospora* populations are vital to protect this species and the complex marine ecosystems it underpins in our increasingly warming oceans.

## Author Contributions

**Rosalie J. Harris:** writing – original draft (equal). **Callum Bryant:** writing – original draft (equal). **Andrea Leigh:** writing – original draft (equal). **Melinda A. Coleman:** writing – original draft (equal). **Adrienne B. Nicotra:** writing – original draft (equal). **Georgina Wood:** writing – original draft (equal).

## Funding

This work was supported by the Australian Research Council (IE230100464) and Holsworth Wildlife Research Endowment.

## Conflicts of Interest

The authors declare no conflicts of interest.

## Supporting information


**Data S1:** ece372720‐sup‐0001‐Supinfo.docx.

## Data Availability

Data are available within the Dryad Database: https://doi.org/10.5061/dryad.q83bk3jw2.
