# Supplementary material for: Thermal Tolerance Varies Latitudinally and Broadly Mirrors Genetic Structure in the Seaweed Phyllospora comosa Across Its Entire Latitudinal Range
Source: Ecol Evol. 2025 Dec 22;15(12):e72720. doi: 10.1002/ece3.72720 (PMC12723070; doi:10.1002/ece3.72720)
Supplement: Supplementary file 1 — Data S1: ece372720‐sup‐0001‐Supinfo.docx. [file ECE3-15-e72720-s001.docx]

## **Supplementary material**

**
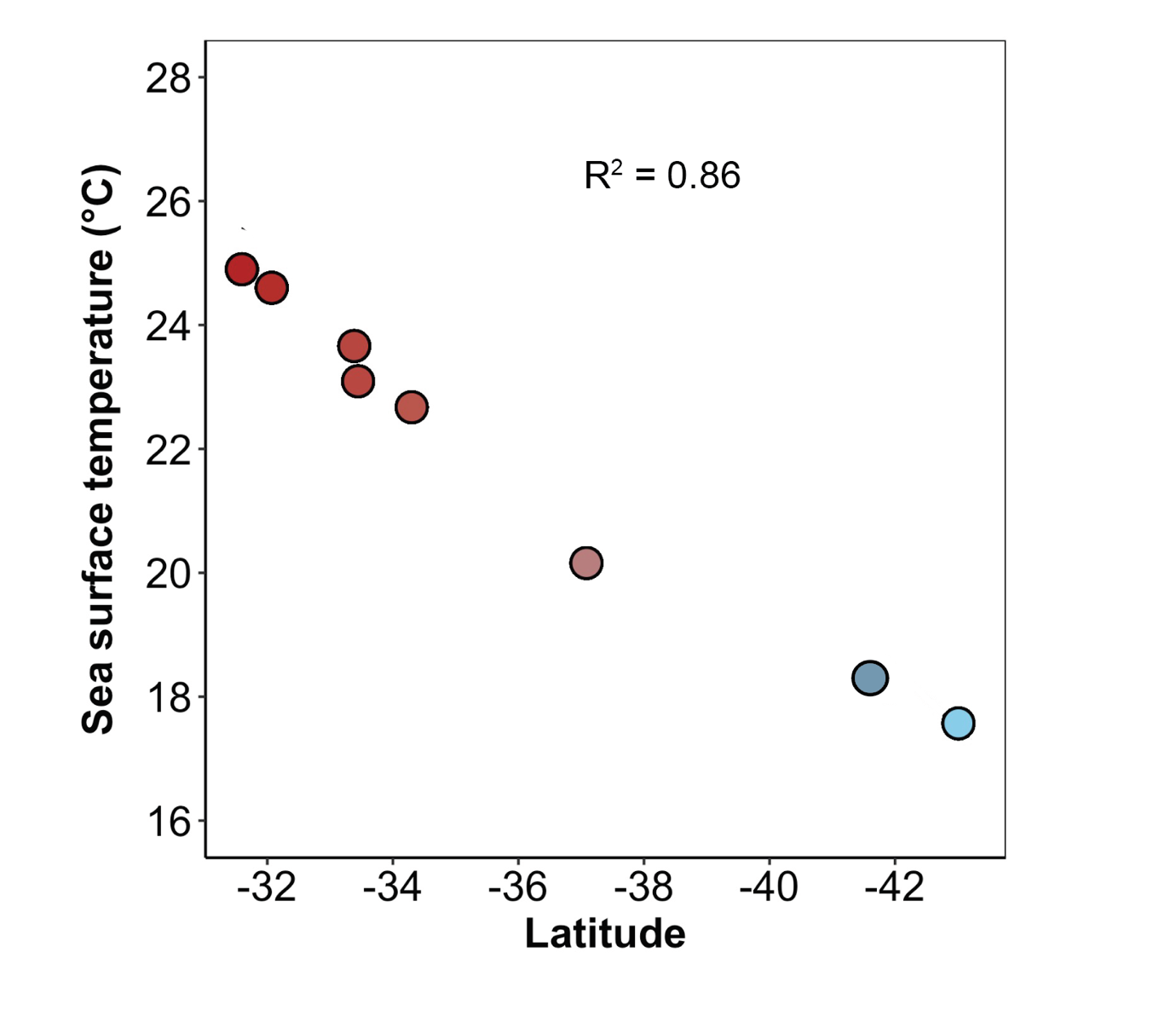
**

**Figure S1.** Temperature profile across latitude for the time of sampling at each site (late February which is the warmest SST). Points are averages from 20 minutes of in-situ temperature logged during collection, points are coloured by changes in latitude.

| **Supplementary Table 1.** Summary of linear mixed-effects model to determine if *T*_crit_ varies across sites from each genetic group (site nested in genetic group). The estimates shown below are the average differences in *T*_crit_ compared to the reference site of Port Macquarie (PM). | | | | |
| --- | --- | --- | --- | --- |
| **Site** | ***Estimate (°C)*** | ***se*** | ***t-value*** | ***P-value*** |
| Intercept: (Port Macquarie (PM)) | 38.172 | 3.266 | 11.686 | **< 0.001** |
| Forster (FO) | -0.239 | 3.925 | -0.061 | 0.951 |
| Bateau Bay (BB) | -0.965 | 4.619 | -0.209 | 0.834 |
| Terrigal (TE) | -4.529 | 4.619 | -0.980 | 0.327 |
| Shark Park (SP) | -2.087 | 4.619 | -0.452 | 0.651 |
| Eden (ED) | -3.679 | 4.619 | -0.797 | 0.426 |
| Bicheno (BI) | -4.569 | 4.619 | -0.989 | 0.323 |
| Port Arthur (PA) | -8.190 | 4.620 | -1.773 | 0.077 |
